# Effect of Psychosocial Interventions on Children and Youth Emotion Regulation: A Meta-Analysis

**DOI:** 10.1007/s10488-024-01373-3

**Published:** 2024-05-08

**Authors:** Kristin Espenes, Anita J. Tørmoen, Kristian Rognstad, Karianne H. Nilsen, Pamela M. Waaler, Tore Wentzel-Larsen, John Kjøbli

**Affiliations:** 1 Center for Child and Adolescent Mental Health, Eastern and Southern Norway, Postbox 4623, 0405 Oslo, Norway; 2 Department of Psychology, University of Oslo, Oslo, Norway; 3 National Centre for Suicide Research and Prevention, University of Oslo, Oslo, Norway; 4 Norwegian Centre for Violence and Traumatic Stress Studies, Oslo, Norway; 5 Department of Education, University of Oslo, Oslo, Norway

**Keywords:** Emotion regulation, Children, Youth, Transdiagnostic, Mental Health

## Abstract

To investigate the effect of psychosocial interventions on emotion regulation outcomes in children and youth (0–23 years). We conducted a meta-analysis using a three-level modeling approach extracting multiple effect-sizes from experimental and quasi-experimental studies. We included 40 interventions from 35 publications involving 3,891 participants and extracted 258 posttreatment effect sizes. Analyses were performed to assess intervention effects on emotion regulation, moderating effects of inclusion of acceptance focus and other potential moderators. Additionally, we examined intervention effects on mental health outcomes. Interventions showed a significant small-to-medium effect on emotion regulation (*d* = 0.37, 95% CI [0.22, 0.51], *p* < .001). Similar effects on were found regardless of acceptance-focus. Interventions significantly associated with a higher level of effect (*p* ≤ 0.045) included ACT, DBT, CBT and behavior parent training interventions. Significant small-to-medium effects were found for mental health outcomes (*d* = 0.39, 95% CI [0.25, 0.53], *p* < .001), with a correlation of .56 between overall outcomes across domains. Meta-regression results indicated that psychosocial interventions are moderately effective in improving emotion regulation, with no significant difference in inclusion of acceptance-focus. Findings suggest that emotion regulation is a transdiagnostic process that may inform the development of more beneficial interventions.

**Supplementary Information:**

The online version contains supplementary material available at 10.1007/s10488-024-01373-3.

In recent years, emotion regulation has been theorized as a transdiagnostic construct that underlies the development and maintenance of diverse forms of psychopathology; consequently, emotion regulation has been framed as a central target of interventions for a range of clinical difficulties across children, adolescents, and adult populations (e.g., Aldao et al., 2016; Compas et al., 2017; Gratz et al., 2015). In this meta-analysis we test how effective psychosocial interventions are at improving child and adolescent emotion regulation outcomes.

Emotion regulation can be broadly defined as the capacity to manage one’s own emotional responses (Young et al., 2019), and more specifically, as goal-directed behaviors to control or modulate emotions, where both automatic and deliberate emotional processes are influenced by the use of strategies (Gross, 2015). Adaptive regulation is understood as context-dependent use of strategies to up-and down-regulate both positive and negative emotions (McRae & Gross, 2020). Expanding the conceptualization of emotion regulation with an clinical-contextual emphasis on the ability to experience a full range of emotional content, Gratz and Roemer (2004) included a focus on emotional awareness, understanding, and acceptance of emotional content, in addition to the ability to engage in goal directed behavior, refrain from impulsive behavior, and access effective regulatory strategies when needed. Thus, a hallmark of adaptive emotion regulation is successfully applying regulating strategies to respond flexibly to changes in one’s external and internal situational demands (Aldao et al., 2015). Conversely, the related concept of emotion dysregulation is reflected in generalized difficulties in these skills, resulting from a failure to regulate emotions when needed or choosing to implement poorly matched strategies (Gross, 2013). In the current review, emotion regulation will refer to abilities or skills in experiencing or modulating emotions, whereas emotion dysregulation will refer to aforementioned difficulties in these domains. Scholars have argued that emotion regulation is a core developmental aspect of human maturation (Cole et al., 2004) and is essential to the development of social competence, academic achievement, and psychological well-being (e.g., Eisenberg et al., 2010; Gross & Muñoz, 1995).

Emotion regulation strategies play a key role in mental health symptoms in both child and adult populations (Aldao et al., 2010; Bender et al., 2012; Nolen-Hoeksema et al., 2008), and has been examined in systematic reviews and meta-analyses (e.g., Aldao et al., 2010; Daros et al., 2021; Moltrecht et al., 2020; Schäfer et al., 2017; Sloan et al., 2017) and across psychopathology categories and comorbidities (McLaughlin et al., 2011, 2014). Different classifications (e.g., Aldao et al., 2010; Daros et al., 2021) distinguishes the strategies into 1) adaptive and/or engagement strategies, involving active engagement with an emotional experience, and 2) maladaptive and/or disengagement strategies, involving attempts to disengage from an emotional experience (see Table S1 in Supplementary materials for details). Adaptive emotion regulation strategies found to buffer against psychopathology include the engagement skills reappraisal, problem solving, and acceptance (Aldao et al., 2010). Conversely, maladaptive strategies linked to increases in overall mental health difficulties, include the disengagement regulation skills avoidance, suppression, and rumination (Aldao et al., 2010).

The use of emotion regulation strategies to modulate emotions has clear developmental shifts, starting from external parental regulation in early childhood, transitioning to a gradual increased reliance on more complex and flexible internal regulation strategies in adolescence and young adulthood (Compas et al., 2017; Weissman et al., 2019). Deficits in emotion regulation during these formative years are associated with a higher probability of developing anxiety and depressive symptoms and an overall risk of comorbid psychopathology (Young et al., 2019). Furthermore, the relation between childhood maltreatment and later psychopathology can in part be explained by increases in emotional reactivity and engagement in maladaptive emotion regulation strategies (Heleniak et al., 2016; Weissman et al., 2019). This aligns with research highlighting that individuals who, by disposition, are highly reactive to emotions, are also more vulnerable to developing a range of mental health problems (Carver et al., 2017; Caspi & Moffitt, 2018), Specifically, emotional dysregulation has been recognized in youth depression (Silk et al., 2003; Young et al., 2019), anxiety disorders (Schneider et al., 2018), deliberate self-harm (Wolff et al., 2019), the development of personality disorders (Matusiewicz et al., 2014), as well as broader internalizing and externalizing problems (Compas et al., 2017).

## Emotion Regulation as a Transdiagnostic Target

There is seemingly a mismatch between the high instance of youth comorbidity and diagnostical complexity and the single-problem focus many existing treatment interventions employ (Weisz et al., 2017a, b). Therefore, an increased interest in transdiagnostic treatment approaches that focus on shared pathological processes has emerged (Barlow et al., 2013; Norton & Paulus, 2016), suggesting that targeting emotion regulation may lead to beneficial outcomes across mental health symptoms. A number of children and youth psychotherapies have incorporated treatment elements that explicitly teach emotion regulation skills. Nevertheless, different modalities focus on different skill sets; while traditional Cognitive Behavioral Therapy (CBT) interventions may emphasize elements of cognitive reappraisal and restructuring, ‘third wave’ psychotherapies have an added emphasis on acceptance-based skills (Young et al., 2019). ‘Third wave’ child and youth interventions include: Dialectical Behavior Therapy for children (DBT-C; Perepletchikova et al. 2017) and adolescents (DBT-A; Rathus & Miller, 2002), acceptance and commitment therapy for adolescents (ACT; Halliburton & Cooper, 2015; Hayes et al., 1999), and the unified protocol for youth (UP; Barlow et al., 2004; Ehrenreich-May et al., 2017). Such acceptance-focused approaches may be of special interest when examining the effects of emotion regulation interventions, as mindfulness and acceptance involve the non-judgmental acceptance of emotions, thoughts, and sensations as they are (Segal et al., 2018), and a willingness to experience troublesome thoughts and feelings without striving to alter, avoid, or control them (Biglan et al., 2008). Nevertheless, there is substantial variability among these interventions in terms of underlying principles and specific techniques employed. By way of illustration, DBT (Linehan, 1993) considers emotion dysregulation as a core dysfunction which is targeted through carefully balancing *both* acceptance-focused and cognitive behavioral techniques and skills delivered through multiple treatment modes such as skills training groups, individual sessions and telephone consultations. In contrast, ACT (Hayes et al., 1999) places a greater emphasis on aligning personal values and behavior through promoting psychological flexibility and acceptance of unwanted emotional or cognitive content. Interventions are more commonly delivered through individual sessions and do not incorporate a specific skills training component. Furthermore, while both treatments incorporates mindfulness-practices, ACT views guided mindfulness as one of several processes aimed at facilitating psychological flexibility, whereas DBT considers mindfulness a core component highlighted across skills modules (e.g. increasing awareness of emotions and behavioral tendencies to reduce impulsive behavior). Additionally, DBT includes specific acceptance-skills in the Emotion Regulation-module (Linehan, 2015b), such as the concept of “radical acceptance”, which teaches clients to accept emotions, thoughts, or circumstances regardless of their painful content (Linehan & Wilks, 2015a).

Despite the aforementioned differences between interventions, all include concepts such as mindfulness and acceptance which are viewed as antithetical to the aforementioned maladaptive strategies (Chambers et al., 2009), and therefore may address clients’ deficits in emotion regulation skills in a new way.

A recent systematic review has both included younger samples along with adult samples (Sloan et al., 2017), and two meta-analyses especially examined emotion regulation outcomes for children and adolescent populations (Daros et al., 2021; Moltrecht et al., 2020). However, none have included participants younger than 6 years, leaving the possible effects for the younger children unexplored. Furthermore, none have included an extended range of mental health difficulties such as externalizing symptoms or behavioral disorders, even though emotion regulation has also been applied to externalizing disorders (see Aldao et al., 2010; McLaughlin et al., 2014; Nolen-Hoeksema et al., 2008,). Additionally, thus far, none have explored multiple effects for both emotion regulation and mental health outcomes utilizing a three-level approach (Assink & Wibbelink, 2016) integrating all calculable effects sizes from multiple levels.

## Review Aims

Although some aspects regarding the usefulness of psychosocial interventions for emotion regulation are known, several limitations in the literature should be addressed. Firstly, there is limited knowledge regarding the interventions’ effectiveness on emotion regulation treatment targets, especially for younger populations. Secondly, despite the purported intention behind acceptance-focused treatment elements, their specific effect is less explored. Therefore, efforts should be made to elucidate the effects in general, and of acceptance-focused treatment elements in particular, on children and youth emotion regulation outcomes. Also, one would assume that if emotion dysregulation is a transdiagnostic dimension of relevance across mental health disorders, then interventions addressing emotion dysregulation would also promote a decrease in related mental health symptoms.

We address these assumptions in the current meta-analysis by 1) examining whether psychosocial interventions for children and youth, regardless of whether they explicitly claim to target emotion regulation, improve emotion regulation outcomes, 2) exploring whether interventions that focus explicitly on acceptance-focused elements are more effective in improving emotion regulation, and 3) examining whether the interventions improve mental health outcomes for children and youths. Additionally, given a substantial amount of heterogeneity in the estimated effects, we 4) explore candidate moderators based on prior meta-analyses (e.g., De Los Reyes et al., 2015; Moltrecht et al., 2020; Schäfer et al., 2017; Weisz et al., 2017b) including participants’ age and gender, outcome measures type, intervention type, informant type, active vs. passive control condition, and incomplete outcome data bias.

## Method

Publications were included if they had outcome measures for both emotion regulation and mental health. We included both experimental and quasi-experimental studies, to cover as much of the literature as possible. In line with the exploratory aim of this study, we included both interventions with and without an explicit focus on emotion regulation, because if emotion regulation is a transdiagnostic factor, we would expect effects in both emotion regulation and related mental health domains regardless of intervention focus. Following from Sloan et al.’s (2017) meta-analysis, we acknowledged the multi-faceted nature of emotion regulation and chose to use an emotion regulation definition which includes the awareness and understanding of emotions (Gratz & Roemer, 2004), in addition to the adaptive and maladaptive emotion regulation strategies, as described by Aldao et al. (2010). Additionally, we based our understanding of emotion regulation measures on Adrian et al. (2011) review of emotion regulation measures, as well as the aforementioned reviews (Aldao et al., 2010; Sloan et al., 2017). This means we included measures of emotion regulation as a) dysregulation (e.g., affect lability) or maladaptive use of strategies (e.g., rumination, worry), and b) an ability for emotional awareness and understanding (e.g. emotion observation) and use of adaptive strategies, including acceptance or mindfulness concepts. We followed PRISMA (Preferred Reporting Items for Systematic Reviews and Meta-Analyses) guidelines for conducting and reporting this study (Page et al., 2021). In addition, we developed and registered a review protocol in PROSPERO prior to our search following the procedures outlined in the Cochrane Handbook for Systematic Reviews (Higgins et al., 2023).

### Inclusion and Exclusion Criteria

We screened for eligible studies in our search results, and identified relevant studies in other reviews based on the following inclusion criteria:

- Population: children and youths (0–23 years mean age) with either clinical levels (i.e., either meeting the criteria of the Diagnostic Statistical Manual of Mental Disorders (APA, 2022), or subclinical symptoms (i.e., defined as indicative of treatment in the present study) of mental health problems. The decision to set the upper mean age cut-off was informed by the United Nations Department of Economic and Social Affairs (2018) definition of youth, which encompasses individuals between the ages of 15 and 24.
- Interventions: psychosocial interventions intended to address psychological symptoms and/or diagnoses. As noted, although our search aimed at emotion regulation interventions, we included studies with or without an explicit focus on emotion regulation, if they included both emotion regulation and mental health outcomes. We searched for references in which emotion regulation terms were specifically mentioned in the title, abstract and/or controlled vocabulary.
- Comparison: all active or passive control or comparison conditions in experimental and quasi-experimental studies, including treatment as usual (TAU) or other active comparators.
- Outcomes: at least one measure of emotion regulation (e.g. Difficulties in Emotion Regulation Scale, DERS), and one symptom measure related to mental health disorders (e.g. Child Behavior Checklist, CBCL). We included self, parental, teacher, and clinician reported outcomes, as well as task-based scores.

We excluded studies that:

- Included special populations (e.g. autism spectrum disorder, intellectual impairment, psychosis).
- Lacked a control or comparison group.
- Included pharmacological treatment only.

We did not exclude studies based on language or publication status.

### Information Sources

A comprehensive literature search was conducted in PsycINFO (Ovid, 1806 – to Present), Medline (Ovid, 1946 – to Present), and the Cochrane Library (start date not specified). The search strategies included both controlled vocabulary and free-text terms related to psychopathology, emotion regulation, and relevant interventions, and were adapted to each database (see Supplemental Materials).

All references were downloaded to the Covidence systematic review software (Covidence Software, Veritas Health Innovation, Melbourne, Australia, available at www.covidence.org) for pairwise abstract and full-text screening, and Risk of Bias (RoB) assessments. The review team screened abstracts and full-texts independently, and when the reviewers were uncertain about an article's eligibility, the full report was obtained, and discrepancies were discussed. If consensus was not reached, authors KE and KR consulted the wider team.

### Study Selection

To ensure that the intervention and control conditions were distinct, we excluded three studies (Bass & Apsche, 2013; Talley, 2013; Suveg et al., 2018) with control interventions that overlapped with treatment conditions in the other included studies. We are aware of the potential overlap between emotion regulation measures and measures assessing psychopathological symptoms, as well as concerns around emotion regulation measures psychometric properties (Mazefsky et al., 2021). We address these challenges in the discussion.

### Data Extraction

After screening for eligibility, study characteristics such as sample size, age, gender, and control and treatment conditions were entered into a data extraction form by two independent reviewers. All data extraction and RoB assessments, including the incomplete outcome data bias rating, were performed in collaboration with Helland et al. (2022). Five of the authors, in addition to two graduate-students identified relevant abstracts in initial searches. The first author reviewed all abstracts included from the search. No indicators of agreement were tracked. Where there was a disagreement regarding the inclusion of a study, the two researchers reviewed the article and discussed its eligibility until an agreement was achieved. Data extraction and Risk of Bias analyses was done independently by two researchers, along with two graduate students trained in meta‐analytic methodology who also coded each study. All data was manually reviewed by the first author and one additional author to check for data entry errors. No indicators of intercoder reliability or agreement were tracked.

The primary moderator examined was the inclusion of an acceptance or mindfulness component (yes or no), as defined by the intervention features readily available in the primary articles. Interventions were classified as acceptance/non-acceptance focused based on whether they included treatment elements related to acceptance and/or mindfulness. Examples include explicit mindfulness practices (e.g., MBCT-C, Unified Protocol and Rumination-focused CBTs inclusion of mindfulness skills to increase present-moment awareness), in addition to other acceptance-focused elements (e.g. ACT and DBT focus on increased acceptance of emotions and thoughts). We additionally explored several other moderating effect variables. Moderating variables were clustered into intervention, study, outcome, and sample characteristics, and all studies were coded by twelve intervention characteristics (see Tables 1, 2, 3, 4) and distinctions were made between a range of described treatment approaches (e.g. CBT, ACT) to illuminate possible moderating effects related to type of intervention. Variables regarding study characteristics were coded by type of control condition and informant, as well as incomplete outcome data bias score from Risk of Bias assessments, as attrition rates may introduce bias if drop-out participants characteristics are different than those remaining. We coded all variations of treatment as usual (e.g. TAU, enhanced TAU) and other treatment interventions as active conditions, and all types of waitlist conditions (e.g. waiting list, assessment only) as passive. We coded informants as either self-report, parental report, clinician report, or other (e.g. teacher/external observer). Regarding outcome measures, all were coded as follows: emotion regulation, externalizing difficulties, internalizing difficulties, other symptom measures (e.g., CGI-S Clinical Global Severity) and general mental health (e.g., Q-LES-Q Quality of life enjoyment and satisfaction questionnaire). Furthermore, all measures except emotion regulation measures were grouped together as a collective outcome category termed ‘mental-health measures.’

**Table 1:** Study characteristics of all studies and measures included in the meta-analysis

Author	Year	Design	N	Intervention	Control condition	Intervention type	Gender	Mean age (range)	Emotion Regulation measure	Mental Health measure
Bentley et al	2017	RCT	138	Single session Unified Protocol (UP)	AO	UP	Both boys and girls	18.3 (18 +)	ERQ; MEAQ	DASS-21; BAS Behavior activation subscale; Q-LES-Q
Betancourt et al	2014	RCT	436	Youth Readiness Intervention (YRI)	WLC	CBT	Both boys and girls	18 (15–24)	DERS	
Burke & Loeber	2016	RCT	252	STOP NOW AND PLAN (SNAP)	TAU	CBT	Boys only	8.5 (6–12.8)	OEQ; SCS	
Cook et al	2019	RCT/QRCT	159/153^a^	Guided Internet Rumination-focused i-RFCBT/unguided Internet Rumination-focused (i-RFCBT)	WLC	RF/RF	Both boys and girls	(18–24)	PSWQ; RRS	PHQ-9; GAD-7
Cotton et al	2020	NRCT	43	Mindfulness-Based Cognitive Therapy for Children (MBCT-C)	WLC	MF	Both boys and girls	13.6 (9–18)	ERC; CAMM	PARS; STAI; CGI-S
Ettelson et al	2002	RCT	25	Cognitive behavioural therapy group treatment (CBT)	WLC	CBT	Both boys and girls		NMR; COPE	CDI; RCMAS; MASQ; TC
Ford et al	2012	RCT	59	Trauma Affect Regulation: Guide for Education and Therapy (TARGET)	ETAU	ERT	Girls only	17.4 (13–17)	NMR	CAPS-CA; TSCC; PTCI; HOPE scale
Fung et al	2016	RCT	19	Based of Learning to Breathe (L2B)	WLC	MF	Both boys and girls	12.7 (12–14)	ERQ	CBCL; YSR
Fung et al	2018	RCT	145	Based of Learning to Breathe (L2B)	WLC	MF	Both boys and girls	13.9 (13–15)	ERQ; EAC; AFQ-Y8; CRSQ	YSR; PSS
Griffiths et al	2019	RCT	53	Mentalization based treatment for adolescents (MBT-Ai)	TAU	PD	Both boys and girls	15.5 (12–18)	DERS	RTSHI; RCADS; RFQY; ECRS; BPFS-C
Hancock et al	2018	RCT	130/125^b^	Acceptance and Commitment Therapy (ACT)/Cognitive Behavioural Therapy (CBT)	WLC/WLC	ACT/CBT	Both boys and girls	11.2 (7–17)	AFQ	ADIS-IV CSR; MASC; CALIS; CHQ
Hoorelbeke et al	2015	RCT	53	Working memory based Cognitive Control Training (CCT)	AC	CT	Both boys and girls		PSWQ; RRS; PANAS	BDI; MASQ; ACS-NL; RS-NL
Idsoe et al	2019	RCT	228	Adolescent Coping with Depression Course (ACDC)	TAU	CBT	Both boys and girls	16.7 (16–20)	ERQ; RRS	CES-D; ATQ; DAS
Jacobs et al	2016	RCT	33	Rumination Focused CBT(RFCBT)	AO	RF	Both boys and girls	15.5 (12–18)	RRS	RADS; CDRS-R
Kaczkurkin et al	2016	RCT	61	Prolonged exposure for adolescents (PE-A)	AC	EX	Girls only	15.3 (13–18)	NMR; STAXI	CPSSI
Kennedy et al	2018	RCT	47	Unified Protocol for Children (UP-C)	AC	UP	Both boys and girls	9.3 (6–12)	ERQ-CA; CEMS	CDI; SCARED
Lackner et al	2016	RCT	22	Neurofeedback therapy (NFT)	TAU	CT	Girls only	(12–18)	Bf-S SRMS; EKF	CDI; BSI
Lee et al	2020	RCT	39	Acceptance and Commitment Therapy (ACT)	WLC	ACT	Both boys and girls	21 (12–45)	AAQ-II	
Lindqvist et al	2020	RCT	76	Affect-focused internet-based psychodynamic therapy (IPDT)	Supportive contact	PD	Both boys and girls	16.6 (15–18)	DERS; SCS-SF	MADRS-S; GAD-7; QIDS-A17-SR
Luby et al	2018	RCT	229	Parent–Child interaction therapy- emotion development (PCIT-ED)	WLC	BPT	Both boys and girls	5.6 (3–6.1)	ERC; MCQ	K-SADS Depression; PFC; CGAS; PECFAS/CAFAS; CGI
McIndoo et al	2016	RCT	34/30^c^	Mindfulness-based therapy (MBT)/Behavioral activation (BA)	WLC	MF/Other	Both boys and girls	19.2	RRS; FFMQ	BDI-II; HRSD; PSS; BAI
Mogoase et al	2013	RCT	42	Concreteness training (CNT)	WLC	CT	Both boys and girls	22.9	RRS; ABS-II	PEQ; AMT; BDI-II
Olson	2018	RCT	60	Aerobic exercise (AE)	AC	Other	Both boys and girls	21.0 (18–30)	RRS	BDI; BAI
Payne	2019	QRCT	117	Family Connections (FC)	WLC	DBT	Both boys and girls	15.0	DERS	DASS-21 depression subscales; BC
Schuppert et al	2009	RCT	106	Emotion Regulation Training (ERT)	TAU	ERT	Both boys and girls	16.1 (14–19)	LPI-ed; MERLC	SCL-90R; BPDSI; YQOL-R; BPDSI-IV; YSR
Schuppert et al	2012	RCT	43	Emotion Regulation Training (ERT)	TAU	ERT	Both boys and girls	16 (14–19)	MERCL	BPDSI; YSR
Schweizer et al	2017	RCT	30	Internet Rumination-focused CBT (i-RFCBT)	Placebo training	CT	Both boys and girls	15.4 (14–18)	CERQ	IOE-r
Shabani et al	2019	RCT	47/47^d^	Acceptance and Commitment Therapy (ACT) + SSRI/Cognitive Behavioural Therapy (CBT) + SSRI	TAU	ACT/CBT	Both boys and girls	14.9 (12–18)	AFQ-Y8; CAMM	CY-BOCS; CDI; VLQ
Smith et al	2015	RCT	112	Stressbuster Computerized CBT (C-CBT)	WLC	CBT	Both boys and girls	(13–16)	CRSQ	MFQ-C; SCARED; SDQ
Topper et al	2017	RCT	167/169^e^	Group Rumination-focused CBT (g-RFCBT) /Internet Rumination-focused CBT (i-RFCBT)	WLC	RF/RF	Both boys and girls	(15–22)	PSWQ; RRS; PTQ	BDI-II; MASQ-D30 Anxious arousal and General distress subscales
Webster-Stratton et al	2011	RCT	99	Incredible Years (YI)	WLC	BPT	Both boys and girls	(4–6)	SCS-P; WFT; WPST	CBCL; CPRS-R; CTRS; ECBI; TRF; Free play Child behavior—Child Deviance
Whiteside	2010	RCT	145	Brief Alcohol Screening and Intervention for College students DBT skills enhanced (DBT-BASICS)	Relaxation CC	DBT	Both boys and girls	18.9 (17–26)	DERS	BDI-II; BAI
Wilkinson et al	2008	RCT	26	Cognitive Behavioral Therapy plus treatment as usual and SSRI (CBT + TAU + SSRI)	TAU	CBT	Both boys and girls	15.0	RDQ	MFQ
Wineman et al	2009	NRCT	47	Dialectical behavior therapy-based journal writing group (DBT)	TAU	DBT	Girls only	(14–18)	TAS-20; DERS	BDI
Yang et. al	2016	RCT	45	Attention bias modification intervention (ABM)	Placebo ABM training	CT	Both boys and girls	14.9 (12–18)	RRS	HAM-D; CES-D; STAI

*ACT* Acceptance and Commitment therapy, *BPT* Behavior Parent training, *CBT* Cognitive Behavioral Therapy, *CT* Cognitive training, *DBT* Dialectical Behavior Therapy, *ERT* Emotion Regulation Therapy, *EX* Exposure therapy, *MF* Mindfulness focused, *PD* Psychodynamic interventions, *RF* Rumination focused, *UP* Unified Protocol. Abbreviations Instruments: *AAQ-II* Acceptance and Action Questionnaire-II, *ABS-II* Attitude and Belief Scale, *ACS-NL* Attentional Control Scale Attention, *ADIS-IV* Anxiety Diagnoses, *ADIS-IV* Clinical Severity Rating, *AFQ* Avoidance and Fusion Questionnaire, *AFQ-Y8* The Avoidance and Fusion Questionnaire for Youth, *AMT* Autobiographical Memory Test, *ATQ* Automatic Thoughts Questionnaire, *BAI* Beck Anxiety Inventory, *BAS* Behavior Activation Scale, *BC* Behavior Checklist, *BDI* Beck Depression Inventory, *Bf-S* Self-Rating Mood Scale, *BPDSI-IV* total score, *BPF* Borderline Personality Features Scale for Children, *BSI* Brief Symptom Inventory, *CAFAS* Child and Adolescent Functional Assessment, *CALIS* Family and Parent Interference, *CAMM* Child and Adolescent Mindfulness Measure, *CAPS-CA* Clinician-administered PTSD scale for children and adolescents, *CBCL* Child Behavior Checklist, *CDI* Children's Depression Inventory, *CDRS-R* Children’s Depressive Rating Scale–Revised, *CEMS* Children's Emotion Management Scales, *CERQ* Cognitive Emotion Regulation Questionnaire, *CES-D* Depression Scale for Adolescents, *C-GAS* Children Global Assessment Scale score, *CHQ* The Child Health Questionnaire, *CGI-S* Clinical Global Severity and Improvement score, *PARS* Paediatric Anxiety Rating Scale, *COPE* Inventory, *SCS-P* The Social Competence Scale – Parent Version, *CPRS* Conners' Parent Rating Scale-Revised, *CPSSI* Child PTST symptom scale interview, *CRSQ* Child Response Styles Questionnaire, *CTRS-R* Conners Teacher Rating Scale, *CY-BOCS* Children's Yale-Brown Obsessive Compulsive Scale, *DAS* Dysfunctional Attitude Scale, *DASS 21* Depression anxiety stress scales, *DERS* Difficulties in Emotion Regulation Scale, *ECBI* Eyberg Child Behavior Inventory, *ECRS* Experiences in Close Relationships Scale, *EKF* Emotional Competence Questionnaire, *EAC* Emotional approach coping scale, *ERC* Emotion regulation checklist, *ERQ* Emotion Regulation Questionnaire, *FFMQ* Five-Facet Mindfulness Questionnaire, *GAD-7* Generalized Anxiety Disorder, *HAM-D* Hamilton Depression Rating Scale, *HOPE* scale, *HRSD* Hamilton Rating Scale for Depression, *IOE-r* Impact of Event scale-revised, *K-SADS-EC* Depression core score, *LPI-ed* Life Problems Inventory emotional dysregulation subscale, *MADRS-S* Montgomery Åsberg Depression Rating Scale, *MASC* Multidimensional Anxiety scale for children, *MASQ* Mood and Anxiety Symptom Questionnaire, *MEAQ* Multidimensional experiential avoidance questionnaire, *MERLC* The Multidimensional Emotion Regulation Locus of control, *MFQ-C and P* Mood and Feelings Questionnaire, My child questionnaire, *NMR* The Negative Mood Regulation Scale, *OEQ* Outcome Expectations Questionnaire, *PANAS* Positive and Negative Affect Schedule Negative affect, *PECFAS* Preschool and Early Childhood Functional assessment, *PEQ* Problem Elaboration Questionnaire, *PHQ-9* Patient Health Questionnaire, *PFC* Preschool Feelings Checklist—Scale Version score, *PSS* Perceived Stress Scale, *PSWQ* Penn State Worry Questionnaire, *PTCI* Posttraumatic Cognitions Inventory, *PTQ* Perservative Thinking Questionnaire, *QIDS-A17-SR* Quick Inventory of Depressive Symptomatology for Adolescents, *Q-LES-Q* quality of life enjoyment and satisfaction questionnaire, *RADS* Reynolds Adolescent Depression Scale, *RCADS* The Revised Child Anxiety and Depression, *RDQ* Responses to Depression Questionnaire, *RCMAS* Revised children's manifest anxiety scale, *RFQY* Reflective Functioning Questionnaire for Youths, *RRS* Rumination Response Style, *RS-NL* Dutch Resilience Scale, *RTSHI* Risk-Taking and Self-Harm Inventory, *SCARED* Screen for Child Anxiety Related Disorders Child and Parent, *SCL-90R* Symptom Checklist-90-Revised, *SCS* Self-Compassion Scale, *SCS-SF* Self-Compassion Scale short-form, *SDQ* Strengths and Difficulties Questionnaire, *STAI* State-Trait Anxiety Index, *STAXI* The State-Trait Anger Expression Inventory; Task Child behavior—Child Deviance, *TC* Thought Checklist, *TAS-20* Toronto Alexithymia Scale, *TRF ASEBA* Teacher Report Form, *TSCC* Trauma Symptom Checklist for Children, *VLQ* The Valued-Living Questionnaire; Wally Feelings Test; Wally Problem Solving Test, *YQOL-R* Youth Quality-of-life Research Version, *YSR-ASEBA* Youth Self- Report

^a^159 participants in Guided i-RFCBT intervention group; 153 participants in Unguided i-RFCBT intervention group. ^b^130 participants in ACT intervention group; 125 participants in CBT intervention group. ^c^34 participants in MBT intervention group; 30 participants in BA intervention group. ^d^47 participants in ACT + SSRI intervention group; 47 participants in CBT + SSRI intervention group. ^e^167 participants in g-RFCBT intervention group; 169 participants in i-RFCBT intervention group

**Table 2:** Effect sizes per study, Emotion Regulation (posttest)

Study	First Author	Year	Intervention	Instrument	ES	**s** ^**2**^
1	Bentley	2017	UP	ERQ Reappraisal	0.26	0.06
				MEAQ	0.21	0.06
2	Betancourt	2016	YRI	DERS	–0.06	0.01
3	Burke	2016	SNAP	OEQ - Remorse	0.33	0.02
				OEQ -Punish	0.36	0.02
				OEQ - Victim	0.31	0.02
				SCS	0.38	0.02
4	Cook	2019	i-RFCBT guided	PSWQ	0.13	0.03
				RRS	0.36	0.03
			i-RFCBT unguided	PSWQ	0.19	0.03
				RRS	0.23	0.03
5	Cotton	2020	MBCT-C	ERC - Lability	–0.19	0.13
				ERC Regulation	–0.10	0.13
				CAMM	0.27	0.13
6	Ettelson	2002	CBT	NMR	1.26	0.25
				COPE - Avoidant coping	–0.60	0.22
				COPE - Problem focused coping	0.47	0.21
7	Ford	2012	TARGET	NMR	0.07	0.09
8	Fung	2016	L2B	ERQ-CA - Reappraisal	–0.17	0.21
				ERQ-CA- Suppression	0.47	0.22
9	Fung	2018	L2B	ERQ-CA- Reappraisal	0.63	0.04
				ERQ-CA- Suppression	0.13	0.03
				EAC - Emotional processing	0.37	0.04
				AFQ-Y82	0.20	0.03
				CRSQ - Rumination	0.62	0.04
10	Griffiths	2019	MBT-Ai	DERS - Nonacceptance	–0.19	0.08
				DERS - Goal difficulties	–0.03	0.08
				DERS - Impulse control difficulties	–0.18	0.08
				DERS - Lack of emotional awareness	0.06	0.08
				DERS - Emotion regulation strategies	–0.08	0.08
				DERS - Lack of emotional clarity	–0.32	0.08
11	Hancock	2016	ACT	AFQ	0.65	0.04
			CBT	AFQ	0.86	0.04
12	Hoorelbeke	2015	CCT	PSWQ	0.07	0.09
				RRS-NL - Brooding	0.23	0.09
				RRS-NL - Reflection	–0.34	0.09
				PANAS - Positive affect	0.13	0.09
				PANAS - Negative affect	–0.59	0.09
13	Idsoe	2019	ACDC	ERQ - Suppression	0.22	0.03
				ERQ - Reappraisal	0.19	0.03
				RRS - Brooding	0.22	0.03
				RRS - Reflection	0.35	0.03
14	Jacobs	2016	RFCBT	RRS	–0.60	0.19
15	Kaczkurkin	2016	PE-A	NMR - Negative Mood Regulation	0.23	0.07
				STAXI - State anger	0.30	0.07
				STAXI - Trait anger	0.43	0.07
				STAXI - Anger Expression	0.17	0.07
16	Kennedy	2018	UP-C	ERQ-CA - Reappraisal	0.81	0.11
				ERQ-CA - Suppression	0.16	0.10
				CEMS - Anger management	0.69	0.11
				CEMS - Sadness management	0.54	0.10
				CEMS - Worry management	0.00	0.10
17	Lackner	2016	NF	Bf-S SRMS	–0.48	0.21
				EKF - Recognition and understanding own emotions	0.64	0.21
				EKF - Recognition and understanding others emotions	0.33	0.20
				EKF - Regulating and controlling own emotions	0.43	0.20
				EKF - Emotional expressivity	0.15	0.20
18	Lee	2020	ACT	AAQ-II	–0.38	0.40
19	Lindqvist	2020	IPDT	DERS	0.79	0.06
				SCS-SF	0.53	0.06
20	Luby	2018	PCIT-ED	ERC - Lability	1.14	0.02
				ERC - Emotion regulation	0.68	0.02
				MCQ - Guilt reparation	0.52	0.02
21	McIndoo	2016	BA	RRS	1.51	0.18
				FFMQ	0.30	0.15
			MBT	RRS	1.14	0.15
				FFMQ	0.60	0.14
22	Mogoase	2013	CNT	RRS	–0.34	0.10
				ABS-II Global Evaluation/Self-Downing subscale	0.64	0.10
23	Olson	2018	AE	RRS - Depression	–0.01	0.07
				RRS - Brooding	–0.3	0.07
				RRS - Reflection	–0.15	0.07
24	Payne	2019	FC	DERS	0.55	0.08
25	Schuppert	2009	ERT	LPI-ed Emotional dysregulation	0.30	0.04
				MERLC	0.16	0.04
26	Schuppert	2012	ERT	MERLC	–0.73	0.17
27	Schweizer	2017	aWMT	CERQ -Adaptive strategies	0.40	0.14
				CERQ - Maladaptive strategies	1.44	0.17
28	Shabani	2019	ACT group + SSRI	AFQ-Y8	1.67	0.12
				CAMM	1.39	0.11
			CBT + SSRI	AFQ-Y8	1.18	0.11
29	Smith	2015	C-CBT	CRSQ	–0.2	0.04
30	Topper	2017	RFCBT Group	PSWQ	0.76	0.03
				RRS	0.60	0.03
				PTQ	0.67	0.03
			RFCBT Internet	PSWQ	0.68	0.03
				RRS	0.59	0.03
				PTQ	0.56	0.03
31	Webster-Stratton	2011	IY	SCS-P - Emotion regulation	1.09	0.05
				SCS-P - Social Competence	0.29	0.04
				SCS-P - Emotion regulation	0.88	0.05
				SCS-P - Social Competence	0,60	0.04
				Wally Feelings Test	2.00	0.06
				Wally Problem Solving Test	0.00	0.04
32	Whiteside	2010	DBT-BASICS	DERS	0.68	0.05
33	Wilkinson	2008	CBT	RDQ - Brooding	0.79	0.19
				RDQ - Reflecting	0.60	0.18
34	Wineman	2009	DBT journal writing	TAS-20	–0.10	0.10
				DERS	0.54	0.10
35	Yang et. al.	2016	ABM	RRS	0.29	0.09

*AAQ-II* Acceptance and Action Questionnaire-II; *AFQ* Avoidance and Fusion Questionnaire; *AFQ-Y8* The Avoidance and Fusion Questionnaire for Youth; *Bf-S* Self-Rating Mood Scale; *CAMM* Child and Adolescent Mindfulness Measure; *CEM*S Children's Emotion Management Scales; *CERQ* Cognitive Emotion Regulation Questionnaire; *COPE* Inventory; *SCS-*P The Social Competence Scale – Parent Version; *CRSQ* Child Response Styles Questionnaire; *DERS* Difficulties in Emotion Regulation Scale; *EKF* Emotional Competence Questionnaire; *EAC* Emotional approach coping scale; *ERC* Emotion regulation checklist; *ERQ* Emotion Regulation Questionnaire; *FFMQ* Five-Facet Mindfulness Questionnaire; *LPI-ed* Life Problems Inventory emotional dysregulation subscale; *MEAQ* Multidimensional experiential avoidance questionnaire; *MERLC* The Multidimensional Emotion Regulation Locus of control; *MFQ-C/*P Mood and Feelings Questionnaire; My child questionnaire; *NMR* The Negative Mood Regulation Scale; *PANAS* Positive and Negative Affect Schedule Negative affect; *PSWQ* Penn State Worry Questionnaire; *RRS* Rumination Response Style; *SCS* Self-Compassion Scale; *SCS-SF* Self-Compassion Scale short-form; *STAXI* The State-Trait Anger Expression Inventory; *TAS-20* Toronto Alexithymia Scale; Wally Feelings Test; Wally Problem Solving Test

**Table 3:** Overall synthesis results

	Effect Size Analyses						Residual Heterogeneity
Outcome	k	n	Cohen’s d	95% CI		*p*	*p*
Outcome Characteristics				**lb**	**hb**		
Emotion regulation	35	102	0.37	0.22	0.51	< 0.001	< 0.001
Mental health measures combined	31	156	0.39	0.25	0.53	< 0.001	< 0.001
Internalizing difficulties	28	85	0.34	0.18	0.50	< 0.001	< 0.001
Externalizing difficulties	5	23	0.56	0.26	0.87	< 0.001	< 0.001
General mental health	10	16	0.50	0.22	0.79	< 0.001	< 0.001
Other symptom measures	14	32	0.39	0.16	0.61	< 0.001	< 0.001

*m* number of individual study interventions, *k* number of unique effect estimates,* p* p value, *CI* confidence interval, *lb* lower bound, *ub* upper bound

**Table 4:** Moderator analyses for emotion regulation

		Effect Size Analyses					Residual Heterogeneity	
Moderator		k	n	Cohen’s d		95% CI	*p*	*p*
Intervention characteristics								
Intervention focus								
Acceptance focus		19	44	0.39	0.20	0.58	< 0.001	< 0.001
Not-acceptance focus		19	58	0.33	0.16	0.50	< 0.001	< 0.001
Intervention type								
CBT-interventions		8	18	0.42	0.13	0.71	0.004	< 0.001
DBT-interventions		3	4	0.47	-0.04	0.98	0.041	< 0.001
ACT-interventions		3	4	0.71	0.22	1.19	0.005	< 0.001
Emotion regulation focused		3	4	-0.04	-0.56	0.49	0.894	< 0.001
UP-interventions		2	7	0.34	-0.20	0.88	0.211	< 0.001
Rumination focused		5	11	0.27	-0.18	0.73	0.235	< 0.001
Mindfulness focused		4	13	0.34	-0.104	0.77	0.133	< 0.001
Psychodynamic based		2	8	0.24	-0.29	0.77	0.379	< 0.001
Exposure therapy		1	4	0.28	-0.45	1.02	0.444	< 0.001
Behavior parent training		2	9	0.77	0.27	1.27	0.003	< 0.001
Cognitive training		5	15	0.25	-0.13	0.62	0.192	< 0.001
Other		2	5	0.20	-0.35	0.75	0.472	< 0.001
Sample characteristics				lb	hb			
Age (continuous)								
Reference age 15, slope		23	70	-0.029	-0.07	-0.01	0.147	< 0.001
Gender								
Girls only		4	12	0.21	-0.20	0.62	0.315	< 0.001
Boys only		1	5	0.36	-0.34	1.05	0.314	< 0.001
Both genders		30	85	0.37	0.22	0.52	< 0.001	< 0.001
Study characteristics								
Informant type								
Youth self		37	88	0.35	0.21	0.49	< 0.001	< 0.001
Parent		5	14	0.38	0.14	0.62	< 0.001	< 0.001
Control type								
Active		17	59	0.32	0.14	0.49	< 0.001	< 0.001
Passive		18	43	0.41	0.20	0.62	< 0.001	< 0.001
Incomplete outcome data bias								
Low		17	50	0.44	0.25	0.63	< 0.001	< 0.001
Unclear		15	47	0.27	0.07	0.47	0.01	< 0.001
High		3	5	0.31	-0.15	0.77	0.178	< 0.001

*m* number of individual study interventions, *k* number of unique effect estimates, *CI* confidence interval, *lb* lower bound, *ub* upper bound

### Methods for Assessing Internal Validity Risk

Risk of Bias in this review was assessed independently by the authors and research assistants. A third senior researcher was consulted when there was a lack of consensus. For randomized controlled trials (RCTs), the Cochrane Collaboration’s Risk of Bias Tool was utilized; for quasi-experimental designs, we used the Cochrane Effective Practice and Organization of Practice (EPOC) Risk of Bias Tool. Risk of Bias for each domain was rated as high (seriously weakens confidence in the effect estimate), low (unlikely to seriously alter the effect estimate), or unclear.

### Effect Measures

We specified the effect measure as Cohen’s *d* for differences between intervention and control conditions posttreatment. This measure presupposes that the number of participants together with means and standard deviations in the intervention and control conditions were available posttreatment; only these studies were included in subsequent analyses. The variance of Cohen’s *d* was estimated by formula 3 in Marfo and Okyere (2019). To ensure that a positive effect measure signified that the intervention was more favorable than the control condition, the Cohen’s *d* sign was flipped if the outcome measure was in the opposite direction.

### Statistical Analysis

As a preparatory analysis, the cases with the information necessary for computing Cohen’s *d* (n = 258) were compared with cases where this information was not available (n = 46). The dependent variable in these analyses was whether this information and relations with the variables were available for the whole data set, and were investigated by logistic regressions using generalized estimating equations (gee) to take clustering within studies into account. Wherever necessary information was unavailable, we used alternative summary statistics to calculate Cohen’s *d* values (e.g. p values and sample sizes, or t-test statistics).

Since there were multiple effects within studies, the data were analyzed by the three-level procedure implemented in the function rma.mv in the R package metafor (A Meta-Analysis Package for R; Viechtbauer, 2010), following the detailed procedure described in Assink and Wibbelink (2016). Separate effect estimates for emotion regulation and mental health outcomes were computed through a moderator analysis where the different types of outcomes were used as a dichotomous moderator, together with a test for heterogeneity. Three levels of random variation were included: between studies, between effects within studies, and between participants for each effect measure. The last, innermost, level was represented by the variance measures of Cohen’s *d* for each effect. Standard deviations for random variation between and within studies were also estimated, together with percentages of random variation in each of the three levels (Cheung, 2014). For each categorical moderator, an overall effect for each category was estimated together with differences in effects between categories. For continuous moderators, an effect for a chosen reference value and a slope was calculated. A test of residual heterogeneity was also included. We additionally included an overall Wald Test for each multi-category categorical moderator. The following moderators were included in the analyses: acceptance-focus, outcome measures, intervention type, informant type, control condition type, participants’ age and gender, and the incomplete outcome data bias score. We additionally investigated whether interventions are more likely to yield stronger effects on mental health when they yield stronger effects on emotion regulation. To this end, we estimated the correlation between overall effect sizes for mental health and emotion regulation, based on estimated variances and covariances between the overall effect sizes.

Logistic regressions for whether information necessary to compute Cohen’s *d* was available, were performed used the R (The R Foundation for Statistical Computing, Vienna, Austria) package gee (Vincent J. Carey, Ported to R by Thomas Lumley, Brian Ripley). For main analyses, a shiny app (Chang et al., 2020) was developed for the procedure described by Assink and Wibbelink (2016). R code for the shiny app is available in the GitHub repository: (Edited for anonymity).

Additionally, we included in the app Marengo and Montag’s (2020) approach for detecting possible publication bias in a three-level model through examining 1) the funnel plot illustrating the correlations between all observed and predicted scores in each study plotted against their standard error and 2) the significance of a modified Egger’s Regression Test (Egger et al., 1997; Marengo & Montag, 2020) computed by including the standard error as a predictor of effect sizes. Thus, the modified Egger’s Regression Test provides an assessment of the asymmetry of the funnel plot and gives an indication of publication bias if less precise studies show higher effect sizes than more precise studies.

## Results

### Study Selection

**Fig. 1:**
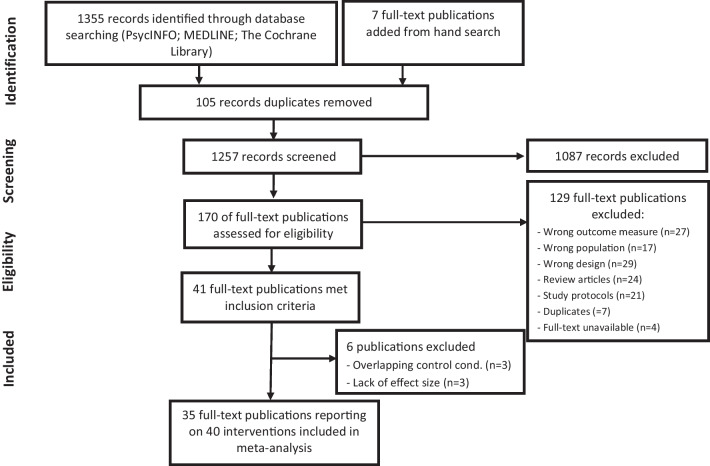
PRISMA flow chart

The PRISMA flow diagram is shown in Fig. 1. An initial literature search was carried out July 6, 2018 and updated May 8, 2020. The two searches produced a total of 1,355 references, where 105 duplicates were removed. Following title and abstract screening, 163 full-text papers were retrieved for further considerations. The first author manually examined all identified review articles for relevant studies, and an additional seven full-text articles were added to full-text screening. After examining the full-texts, 129 articles were excluded. After author correspondence, all included studies provided the data necessary for estimating the effect and variance measures.

### Study, Sample, and Intervention Characteristics

In total, 40 interventions from 35 separate publications (*N* = 3,891 participants) and 258 effect-sizes, whereas 102 reported on emotion regulation outcomes, were included in the final meta-analysis. Included study characteristics were: author(s), publication year, study design, age (mean and range), gender composition, intervention and control condition, emotion regulation or mental health measure, and the Incomplete outcome data Bias Assessment.

The coded articles were published from 2002 to 2020. The majority of the primary studies included participants between 3 and 23 years old (*M* = 15.5 years), though three studies included some participants older than 25 (Olson, 2018; Lee et al., 2020; Whiteside, 2010). In all studies the sample mean age was below 23 years. Sample sizes ranged from 19 to 436 participants. Thirty-one studies (88%) were conducted using an RCT design, one of the RCTs also implemented a mixed randomized-quasi-randomized design, two studies had a quasi-randomized design, and two other studies were non-randomized controlled trials. For studies with multiple treatment conditions where both treatment conditions were assumed to address emotion regulation, both groups were coded and included in the analysis. Twenty studies (56%) used an active control design (i.e. TAU); 15 studies (44%) used a passive control design (i.e. waitlist control). The most commonly employed interventions were variations of CBT (*n* = 8; 24%). Thirty studies included both genders in the treatment samples (82%), four studies included only girls, and one study included only boys.

#### Risk of Bias Assessment Within and Across Studies

**Fig. 2:**
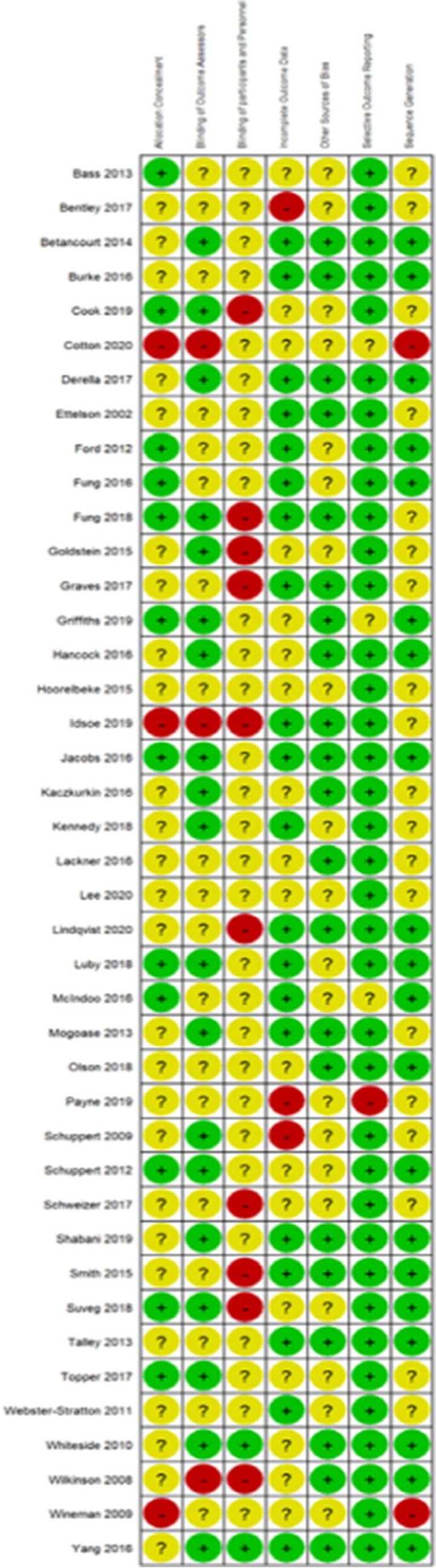
Risk of bias assessment within studies

**Fig. 3:**
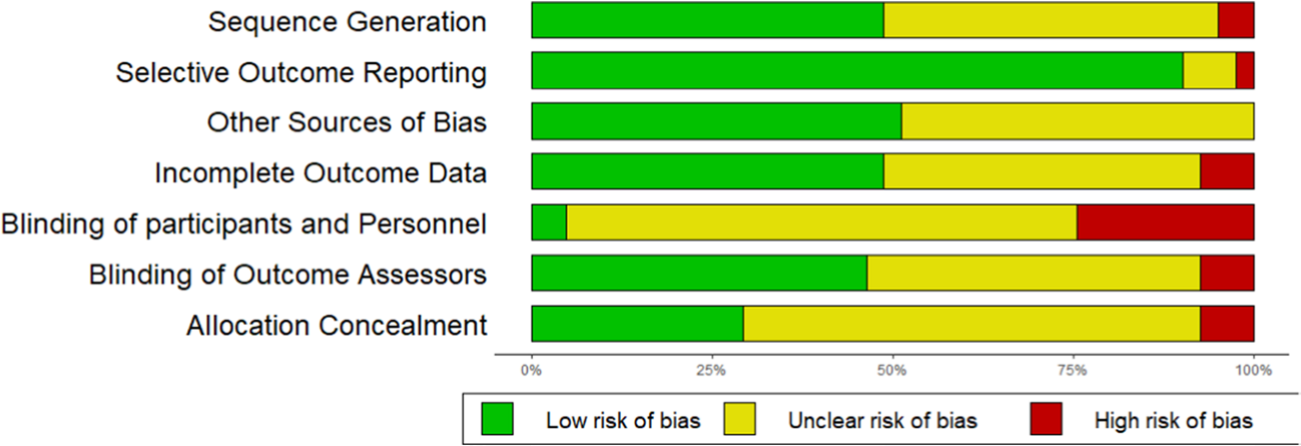
Risk of bias assessment across studies

Figures 2 and 3 illustrate the overall risk of bias within and across studies. All 43 interventions evaluations fulfilling the search criteria were assessed, including interventions with control conditions that overlapped with treatment conditions in the other included interventions. Allocation concealment was not sufficiently described in three studies (Cotton et al., 2020; Idsoe et al., 2019; Wineman, 2009), 26 studies were unclear, and the remaining 12 studies were deemed as low risk of bias. Two studies did not report on sequence generation (Cotton et al., 2020; Wineman, 2009), 19 studies had an unclear sequence generation, and the remaining 20 studies were deemed as low risk of bias. Only two studies (Whiteside, 2011; Yang et al., 2016) reported blinding of participants and personnel, 10 studies were deemed as high risk, and the remaining 29 studies unclear risk of bias. Nineteen studies reported that the outcome assessor was blinded, three studies were high (Idsoe et al., 2019; Wilkinson & Goodyer, 2008; Cotton et al., 2020), and 19 studies were unclear risk of bias. As for the incomplete outcome assessment, only three studies (Bentley et al., 2017; Payne, 2019; Schuppert et al., 2009) were assessed as high risk due to a high dropout-rate and missing data; 19 studies were assessed as low, and 18 as unclear risk of bias. Selective outcome reporting of outcome measures was detected in only one study (Payne, 2009), three studies were unclear, and 37 were assessed as low risk of bias. No high risks in other sources of bias were detected.

A summation of the overall risk of bias across studies (Fig. 3) shows that the majority of information came from trials with low or unclear risk of bias. An exception was the blinding of participants and personnel where a higher proportion of information (about 20%) was assessed as high risk of bias.

### Synthesis Results

There was significant heterogeneity (*Q* = 1201.588, *df* = 257, *p* < 0.001) among the overall effect sizes in the full dataset when we compared study interventions with any control condition. Furthermore, the Likelihood Ratio Test, where we distinguished between 258 unique effect sizes (level 2) clustered in 40 interventions in 35 studies (level 3), showed that significant variance was present at both the within (level 2: *SE* = 0.42, *p* < 0.001) and between study level (level 3: *SE* = 0.30, *p* < 0.001). This suggest that there is room for within-and between-study characteristics that may impact on the overall effect.

### Meta-Analysis

#### Emotion Regulation

We examined the intervention effect on emotion regulation outcomes (*n*_emotion regulation_ = 102, see Table 2) through a moderation analysis with a dichotomous moderator for emotion regulation versus mental health outcomes, and found a significant posttreatment effect size of Cohen’s *d* = 0.37, 95% CI [0.22, 0.51], *p* < 0.001 which suggests that the interventions improve emotion regulation when compared to control conditions. The test for heterogeneity on the dataset including only emotion regulation outcomes revealed a reduced, but still significant value (*Q* = 301.232, *df* = 101, *p* < 0.001), with significant variance between studies (level 3: *SE* = 0.33, *p* < 0.001), but not at the within study level (level 2: *SE* = 0.15, *p* < 0.001).

#### Emotion Regulation and Acceptance-Focused Interventions

We furthermore used the reduced dataset with only the emotion regulation effects sizes to examine whether acceptance-focused interventions were more effective in improving emotion regulation than non-acceptance focused interventions (*n*_acceptance_ = 44; *n*_non-acceptance_ = 58). Cohen’s *d* for acceptance-focused interventions was *d* = 0.39, 95% CI [0.20, 0.58], *p* < 0.001, and for non-acceptance focused a Cohen’s *d* = 0.33, 95% CI [0.16, 0.50], *p* < 0.001, which gives an estimated difference in Cohen’s *d* for non-acceptance versus acceptance focused interventions of -0.06 95% CI [-0.30, 0.18], *p* = 0.621. The result is consistent with an effect on emotion regulation outcomes of similar magnitude regardless of acceptance-focus.

#### Mental Health

Furthermore, we examined intervention effects on the collective mental health domain (*n*_mental health_ = 156) through a moderation analysis with a dichotomous moderator for emotion regulation versus mental health outcomes and found a significant effect size of Cohen’s *d* = 0.39, 95% CI [0.25, 0.53], *p* < 0.001, suggesting an intervention effect on mental health symptoms of similar magnitude to the effect on emotion regulation. Indeed, the estimated difference for mental health versus emotion regulation measures when examining the overall dataset was negligible with a Cohen’s *d* difference of 0.022, 95% CI [-0.11, 0.16], *p* = 0.746. We additionally examined the intervention effects for the specific types of outcome measures, and found significant effects which were slightly higher for externalizing difficulties (*d* = 0.56, 95% CI [0.26, 0.87], *p* < 0.001) and general mental health measures (*d* = 0.50, 95% CI [0.22, 0.79], *p* < 0.001), than for internalizing difficulties (*d* = 0.34, 95% CI [0.18, 0.50], *p* < 0.001) and other symptom measures (*d* = 0.39, 95% CI [0.16, 0.61], *p* < 0.001). These results may suggest that intervention effects are stronger for externalizing and general mental health outcomes, and to a lesser extent, internalizing symptoms.

#### Correlations between Emotion Regulation and Mental Health

We used all eligible studies with emotion regulation and mental health outcomes (32 studies; *n*_emotion regulation_ = 98, *n*_mental health_ = 156) to investigate whether interventions are more likely to yield stronger effects on mental health when they yield stronger effects on emotion regulation. We found that a correlation of 0.56, indicating that a high overall effect of emotion regulation is related to a high overall effect in mental health.

#### Other Moderator Analyses

As there was a substantial amount of heterogeneity in the estimated effect sizes, analyses were performed to identify the potential impact of prespecified moderators on emotion regulation outcomes. Results are presented in Table 4.

We examined the moderators of sample, study and intervention effect. Findings indicated that the following moderators were significantly associated with a higher level of effect on emotion regulation posttreatment (*p* ≤ 0.045): ACT, DBT, CBT and behavior parent training interventions, youth or parent informant, active and passive control condition, samples including both genders, and low and unclear risk of incomplete outcome data bias. Other intervention types (i.e. rumination focused, psychodynamic, cognitive training), samples restricted to either boys or girls, and high risk of incomplete outcome data bias were not significant associated with higher levels of effect on emotion regulation posttreatment (*p* ≥ 0.444). All moderators showed a residual heterogeneity with p < 0.001, indicating heterogeneity in the data even after taking each moderator into account.

#### Publication Bias

**Fig. 4:**
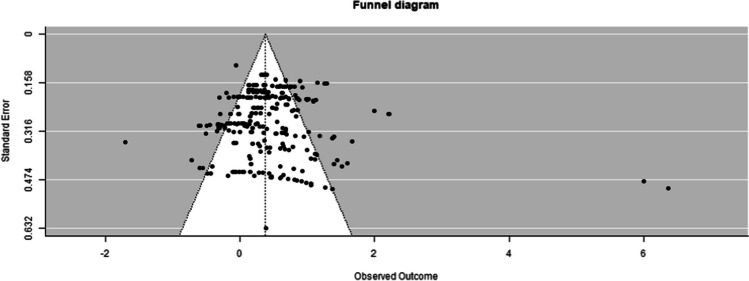
Publication bias funnel plot

The investigation of publication bias was completed via funnel plot visualization (see Fig. 4) and a modified Egger’s Test (Egger et al., 1997; Marengo & Montag, 2020). The distribution of effect sizes was mostly symmetrical with only a few effects sizes in the outer range, which suggests no substantial publication bias. However, the modified Egger’s Test pointed to a significant positive relationship of the effect size with the standard error (*B* = 3.82, 95% CI [2.67, 4.97], *p* < 0.001). An additional sensitivity analysis was conducted where two parental report measure effects from Webster-Stratton et al. (2011) were excluded. This analysis demonstrated a high p value (*p* = 0.182), indicating that these two effects substantially contributed to the perceived publication bias.

## Discussion

This comprehensive systematic review and three-level meta-analysis examined the effectiveness of psychosocial interventions on emotion regulation outcomes among children and youth. In line with the existing literature (Daros et al., 2021; Moltrecht et al., 2020; Sloan et al., 2017), we found a significant small-to-medium effect size on child and youth emotion regulation (*d* = 0.37). Combined with previous findings showing that targeting emotion regulation processes in treatment produces beneficial outcomes across mental health difficulties (e.g., Linehan, 1993; Hayes et al., 1999; Barlow et al., 2010), the findings supports the notion that emotion regulation is an underlying transdiagnostic process. Furthermore, with the advancing addition of acceptance and mindfulness treatment elements in ‘third-wave interventions’ to enhance treatment effects (Chambers et al., 2009), we examined whether this was reflected in different moderating effects on emotion regulation. We found a significant small-to-medium effect size regardless of acceptance-focus *(d* = 0.33 and 0.39), with no significant difference between them. One possible interpretation could be that interventions derived from more traditional models without an explicit focus on acceptance may also effectively address emotion regulation, albeit with a difference in semantics, as they may explicitly aim to reduce the use of maladaptive strategies (Chambers et al., 2009). If so, this may in part explain the lack of significant effect differences between acceptance-focused and other interventions included in the current review.

Furthermore, since emotion regulation processes cut across symptom categories, and are considered a predictor of various forms of psychopathology (e.g., McLaughlin et al., 2011) we expected to see this reflected in concomitant improvement in mental health outcomes (see Aldao et al., 2016). We found a significant small-to-medium overall effect size indicating that the interventions effectively improved mental health outcomes (*d* = 0.39). Although the similarity of the emotion regulation and the mental health estimates could be interpreted as questioning whether these constructs are truly different, we would argue that the confidence interval for this difference (CI [-0.11, 0.16]), indicate that a substantial difference cannot be excluded either. Additionally, similar to previous studies (Moltrecht et al., 2020) we found a strong correlation between high overall effects of emotion regulation and high overall effects in mental health. Following from the literature on regulation difficulties as a predictor for a wide range of psychopathology (e.g., McLaughlin et al., 2014; Young et al., 2019), we suggest that due to its transdiagnostic nature, emotion regulation is potentially a more proximal outcome than mental health (Aldao et al., 2016). Thus, interventions would be expected to produce recognizable effects across outcome domains.

### Conceptual and Clinical Implications

This review has several conceptual and clinical implications. While emotion regulation processes in child and youth samples have been previously examined (Sloan et al., 2010; Moltrecht et al., 2020; Daros et al., 2021), this review fills an important literature gap by reporting on multiple posttreatment effects for emotion regulation, as well as on a broader range of mental health symptoms, and including younger samples (under 6 years old). Although there are similarities between the current review and existing literature, there is limited overlap in of included studies across the three meta-analyses (31.43% overlap with Daros et al., 2021; 11.43% overlap with Moltrecht et al., 2020; 19.05% overlap between Daros et al., 2021 and Moltrecht et al., 2020). One single study (Jacobs et al., 2021) was included in all three meta-analyses. This may be a reflection of minor disparities in specific search criteria and inclusion criteria employed in the respective studies. The search criteria for the current review requested that emotion regulation terms were explicitly included in the title, abstract and/or controlled vocabulary, thus somewhat limiting the scope of the search (1257 studies). Daros et al. (2021) conducted a two-part, broader search, resulting in the inclusion of 88 studies and 385 effect sizes, which is a substantial number. Conversely, but similarly to the current review, Moltrecht et al. (2020) based the search on criteria outlined in Sloan et al., (2017), resulting in a more limited number of studies (21). Moreover, it is noteworthy that the inclusion criteria of Daros et al. (2021) focused on youth and young adults (14–24 years), whereas Moltrecht et al. (2020) included child populations (6–23 years) but excluded younger populations (0–6 years) which diverges from our study. Additionally, similar to our review, Daros et al. (2021) included subclinical populations, while Moltrecht et al. (2020) used diagnostic criteria and symptoms which may have limited their search results. Furthermore, we would argue that the conceptualization of emotion regulation and dysregulation exhibits sufficient similarities, thus the current review did not distinguish between emotion regulation and dysregulation measures in our analyses.

As the aforementioned reviews, we argue that this meta-analysis supports the notion of emotion regulation as a transdiagnostic treatment construct. This is partly due to our finding that interventions had a positive effect on not just emotion regulation, but also mental health outcomes, indicating that changes occur in both constructs following treatment. Additionally, the interventions found in our search that did not explicitly include acceptance-based strategies (e.g., CBT, behavioral parent training), but still utilized emotion regulation outcome measures, also reported beneficial outcomes. Moving forward, research should further examine correlations between changes in emotion regulation and mental health outcomes, and whether emotion regulation mediates effects on mental health outcomes (Heleniak et al., 2016; Weissman et al., 2019). This may be especially crucial for comorbid populations, as further intervention development and refinement may provide improvement across symptoms. To date, this has been minimally explored and research is too scarce to provide clarity (see Moltrecht et al., 2020).

While this study could not ascertain whether or not an explicit focus on acceptance yielded different effects, it is noteworthy that the state of research pertaining to interventions for children and youths is still in its infancy. Currently, there is a dearth of knowledge regarding which treatment elements are most effective, or the appropriate developmental stages for their implementation. Notably, moderator analyses suggested that certain “third wave” interventions such as ACT and DBT were associated with a high level of effect on emotion regulation outcomes, which may imply that they contain elements especially relevant for emotion regulation targets.. Moving forward, the further integration of carefully selected effective elements from traditional CBT-interventions (e.g. cognitive reappraisal) and ‘third wave’ acceptance-focused elements (e.g. radical acceptance) might result in more effective interventions.

### Limitations

Our limitations naturally reflect dilemmas associated with the meta-analytic process (e.g. creating comparison groups by collapsing categories), and the broad range of interventions and outcomes in our included studies. This calls for caution when interpreting our results. Firstly, our estimates and inferences are limited by the studies sampled, despite our three-level approach that enabled the inclusion of multiple outcomes, which would have otherwise been discarded using traditional meta-analytic procedures. Still, the results were based on a relatively limited number of studies because of the scarcity of existing research that has included measures addressing both emotion regulation and mental health difficulties for younger populations. Additionally, limitations related to our search strategy could have affected the number of identified relevant studies. Given the vast array of potential search terms that could have been included, the decision to adhere to a focused search strategy may have resulted in the omission of relevant literature, thereby impacting the comprehensiveness of our review. Further exploration into a wider selection of search terms, such as those aimed at infant or young child populations (e.g., temperament), or expanding the scope to more explicitly encompass constructs such as emotion lability and understanding of emotions could have enhanced the inclusivity of our study and provided a more comprehensive understanding of the research landscape. Regarding study samples, although studies with small sample sizes are fairly common in meta-analyses, more restricted samples can lead to more uncertainty in result interpretation. Overall, this highlights the need for more robust study designs with larger samples.

Furthermore, despite the advantages of close collaboration in this study, the lack of a standardized coding framework may have resulted in variability in how coders interpreted and categorized intervention characteristics, particularly in distinguishing between acceptance and non-acceptance interventions. This variability could potentially raise concerns regarding the reliability and validity of the study's findings, for example the lack of significant effect differences between acceptance-focused and other interventions.

**An additional** central limitation lies in the diversity of terminologies employed for the emotion regulation construct. Consequently, research has found varying psychometric properties of emotion regulation measures (Mazefsky et al., 2021), implying discrepancies in what underlying concept is being measured. An additional limitation stems from the potential overlap between assessment measure items for emotion regulation strategies and psychopathology, especially since affective facets may be captured in both constructs (Aldao et al., 2010). Even though some relations between emotion regulation strategies and specific psychopathology (e.g. between rumination and depression; Nolen-Hoeksema et al., 2008) have been investigated, further research should continue to explore potential confounding relations.

Lastly, the Risk of bias assessments suggest that, although the number of included studies characterized by high risk of bias was small, a great proportion were associated with unclear risk of bias partly due to poor reporting. In our study, the risk of bias in domains such as incomplete outcome data and sequence generation was considerable. Additionally, it is important to acknowledge the lack of inter-rater reliability coefficients for included moderators, which serves as a significant limitation when interpreting the findings.

#### Suggestions for Future Research

It is essential that future effectiveness studies include emotion regulation outcome measures, so research can further explore how emotion regulation is related to mental health difficulties, both in studies of traditional CBT-interventions and especially in ‘third wave’ interventions that claim to target emotion regulation. Furthermore, as Sloan et al. (2017) highlights, it is not clear whether interventions that improve emotion regulation simultaneously produce reductions in numerous comorbid symptoms as too few effectiveness studies have examined outcome measures for multiple psychological disorders. Future research should include controlled study designs with various diagnostic groups where changes in emotion regulation and mental health are examined across time points during treatment to more precisely define when changes occur. In summation, the transdiagnostic quality and mediating capabilities of emotion regulation is still to be determined (Aldao et al., 2016).

Furthermore, we argue that emotion regulation lacks conceptual clarity, reflected in the need for refinement of the vast array of measures used to examine emotion regulation in younger populations (Adrian et al., 2011) as wells as concerns of the psychometrics properties of emotion regulation measures (Mazefsky et al., 2021). Future research should allow for a more precise differentiation between emotion regulation aspects, as well as a discernment of related emotion regulation and psychopathology constructs.

To our knowledge, an overarching protocol on how to effectively target emotion regulation during treatment does not yet exist; therefore, each treatment element’s effectiveness across age groups, clinical populations and treatment criteria is still undetermined. In extension, more knowledge is needed regarding how elements work for different age groups, especially considering the developmental shifts that occur in managing emotional responses and using concrete emotion regulation strategies (Adrian et al., 2011). This calls for future research that utilizes experimental designs to identify change processes that assess the effects of explicit treatment elements on specific outcomes, as well as its optimal dosage. Through dismantling trials, along with factorial or time series experiments, it may be possible to identify the active elements in existing treatment protocols. This knowledge may be helpful in optimizing existing interventions and tailoring individual treatment courses based on the optimal combination and dosage of effective elements.

## Conclusion

We extend the emotion regulation and psychopathology literature by providing, to our knowledge, the first three-level meta-analysis that reports psychosocial interventions to have meaningful effects on emotion regulation and mental health outcomes in children and youths. Our findings additionally expand on previous meta-analyses and existing literature (e.g. Daros et al., 2021; Moltrecht et al., 2020; Sloan et al., 2017), by including younger participants and examining outcomes in a broader range of mental health difficulties. Although research is expanding our understanding on the role of emotional processes across psychopathology, there is still an essential need to improve clinical care for children and their families. More research on emotion regulation as a transdiagnostic process may help optimize future interventions.

## Supplementary Information

Below is the link to the electronic supplementary material.

Supplementary file1 (DOCX 18 KB)

Supplementary file2 (DOCX 25 KB)

Supplementary file3 (DOCX 32 KB)

## Data Availability

A semicolon-separated csv file for the data on which the three-level analyses were based is available on GitHub: https://github.com/ToreWentzel-Larsen/threelevel.
