# Supplementary material for: Effect of Psychosocial Interventions on Children and Youth Emotion Regulation: A Meta-Analysis
Source: Adm Policy Ment Health. 2024 May 8;52(5):833–52. doi: 10.1007/s10488-024-01373-3 (PMC12449357; doi:10.1007/s10488-024-01373-3)
Supplement: Supplementary file 1 — Supplementary file1 (DOCX 18 KB) [file 10488_2024_1373_MOESM1_ESM.docx]

| Classification | Relation to Psychopathology | Strategy | Overview |
| --- | --- | --- | --- |
| Maladaptive/Dis-engagement ER skills | Positively associated with psychopathology | Avoidance | A generalized behavioral or cognitive response to evade experiences perceived as anxiety-provoking or distressing. Encompasses both behavioral (situations, people or events) and experiential (thoughts, feelings, memories and physical sensations) avoidance. |
|  |  | Suppression | Attempts to decrease or suppress unhelpful or undesirable thoughts and/or emotional expressions of an emotional experience. |
|  |  | Rumination | Passive and repetitive focusing of the causes and consequences of emotional experiences; excessive attention to symptoms related to emotional distress or negative mood. |
|  |  |  |  |
| Adaptive/Engagement ER skills | Negatively associated with psychopathology | Cognitive reappraisal | Changing the emotional impact of a situation through neutral or positive reinterpretations of its meaning. |
|  |  | Acceptance | The ability to experience a present-moment awareness and non-judgement of feelings, thoughts and sensations. Closely related to the mindfulness-construct. |
|  |  | Problem-solving | The conscious attempts to change a situation or its effects through solution generation and implementation. |

**Table S1**

*Overview of Emotion Regulation Strategies*

Based on Aldao et al., 2010; Aldao et al., 2016; Daros et al., 2021; Schäfer et al., 2017; Sloan et al., 2017.
