# Supplementary material for: Effect of Psychosocial Interventions on Children and Youth Emotion Regulation: A Meta-Analysis
Source: Adm Policy Ment Health. 2024 May 8;52(5):833–52. doi: 10.1007/s10488-024-01373-3 (PMC12449357; doi:10.1007/s10488-024-01373-3)
Supplement: Supplementary file 2 — Supplementary file2 (DOCX 25 KB) [file 10488_2024_1373_MOESM2_ESM.docx]

**Table S2 Search strategy**

#1          ((affect* or emotion*) near/2 (regulat* or dysregulat*)) ;ti,ab

#2          ((experiential* or emotion* or behavio* or expressive or thought*) next (avoid* or suppres*)):ti,ab

#3          (emotion* near/1 (manage* or competenc*)):ti,ab

#4          MeSH descriptor: [Rumination, Cognitive] explode all trees

#5          ruminat*:ti,ab

#6          #1 or #2 or #3 or #4 or #5

#7          MeSH descriptor: [Anxiety Disorders] explode all trees

#8          MeSH descriptor: [Borderline Personality Disorder] explode all trees

#9          MeSH descriptor: [Self-Injurious Behavior] explode all trees

#10        MeSH descriptor: [Bipolar and Related Disorders] explode all trees

#11        MeSH descriptor: [Disruptive, Impulse Control, and Conduct Disorders] explode all trees

#12        MeSH descriptor: [Feeding and Eating Disorders] explode all trees

#13        MeSH descriptor: [Mood Disorders] explode all trees

#14        MeSH descriptor: [Substance-Related Disorders] explode all trees

#15        (depress* or anxiety or panic* or borderline or bpd or bipolar* or manic or (self next (harm* or injur*)) or dsh or nssi or suicid*):ti,ab

#16        ((opposition* or defiant or conduct*) near/2 disorder*):ti,ab

#17        (disruptive near/4 disorder):ti,ab

#18        ((eating next disorder*) or anorexia or anorect* or bulimi* or ((purge or binge) near/3 disorder*)):ti,ab

#19        ((drug* or alcohol* or substance* or cannabis or mari?uana or heroin or opiate* or amphetamine* or stimulant*) near/1 (abuse* or depend* or addict* or "use")) .ti,ab

#20        #7 or #8 or #9 or #10 or #11 or #12 or #13 or #14 or #15 or #16 or #17 or #18 or #19

#21        MeSH descriptor: [Psychotherapy] explode all trees

#22        (psychotherap* or psycho-therap*):ti,ab

#23        ((behavio* or cognitive or meta-cognitive or metacognitive or acceptance* or dialectical* or schema* or emotion* or affect* or mindfulness* or compassion*) near/2 (therap* or treatment* or training* or program*)):ti,ab

#24        ((emotion* or affect*) near/1 regulation near/2 (training or therap* or treatment* or program*)):ti,ab

#25        (CBT or DBT or ACT or MCT or "unified protocol"):ti,ab

#26        #21 or #22 or #23 or #24 or #25

#27        (child* or young or youth* or adolesc*):ti,ab

#28        #6 and #20 and #26 and #27
