# Supplementary material for: Effect of Psychosocial Interventions on Children and Youth Emotion Regulation: A Meta-Analysis
Source: Adm Policy Ment Health. 2024 May 8;52(5):833–52. doi: 10.1007/s10488-024-01373-3 (PMC12449357; doi:10.1007/s10488-024-01373-3)
Supplement: Supplementary file 3 — Supplementary file3 (DOCX 32 KB) [file 10488_2024_1373_MOESM3_ESM.docx]

**Table S3 References of included studies**

References of studies included in the systematic review. All studies included in the meta-analysis are marked with an asterix.

1. Bass, C. K., & Apsche, J. A. (2013). Mediation analysis of mode deactivation therapy (reanalysis and interpretation). *International Journal of Behavioral Consultation and Therapy, 8*(2), 1.
2. *Bentley, K. H., Boettcher, H., Bullis, J. R., Carl, J. R., Conklin, L. R., Sauer-Zavala, S., . . . Barlow, D. H. (2017). Development of a Single-Session, Transdiagnostic Preventive Intervention for Young Adults at Risk for Emotional Disorders. *Behavior Modification*, 145445517734354. doi:[https://dx.doi.org/10.1177/0145445517734354](https://doi.org/10.1016/j.jocrd.2019.04.003)
3. *Betancourt, T. S., McBain, R., Newnham, E. A., Akinsulure-Smith, A. M., Brennan, R. T., Weisz, J. R., & Hansen, N. B. (2014). A Behavioral Intervention for War-Affected Youth in Sierra Leone: A Randomized Controlled Trial. *Journal of the American Academy of Child & Adolescent Psychiatry, 53*(12), 1288-1297. doi:10.1016/j.jaac.2014.09.011
4. *Burke, J. D., & Loeber, R. (2016). Mechanisms of behavioral and affective treatment outcomes in a cognitive behavioral intervention for boys. *Journal of Abnormal Child Psychology, 44*(1), 179-189. doi:[http://dx.doi.org/10.1007/s10802-015-9975-0](https://doi.org/10.1016/j.brat.2015.12.012)
5. *Cook, L., Mostazir, M., & Watkins, E. (2019). Reducing Stress and Preventing Depression (RESPOND): Randomized Controlled Trial of Web-Based Rumination-Focused Cognitive Behavioral Therapy for High-Ruminating University Students. *Journal of Medical Internet Research, 21*(5), e11349. doi:[https://dx.doi.org/10.2196/11349](http://dx.doi.org/10.1002/cpp.637)
6. *Cotton, S., Kraemer, K. M., Sears, R. W., Strawn, J. R., Wasson, R. S., McCune, N., . . . Delbello, M. P. (2020). Mindfulness-based cognitive therapy for children and adolescents with anxiety disorders at-risk for bipolar disorder: A psychoeducation waitlist controlled pilot trial. *Early intervention in psychiatry, 14*(2), 211-219. doi:[https://dx.doi.org/10.1111/eip.12848](http://cochranelibrary-wiley.com/o/cochrane/clcentral/articles/675/CN-01297675/frame.html)
7. Derella, O. J., Johnston, O. G., Loeber, R., & Burke, J. D. (2017). CBT-Enhanced Emotion Regulation as a Mechanism of Improvement for Childhood Irritability. Journal of Clinical Child & Adolescent Psychology, 1-9. doi:https://dx.doi.org/10.1080/15374416.2016.1270832
8. *Ettelson, R. G. (2003). The treatment of adolescent depression. *Dissertation Abstracts International: Section B: The Sciences and Engineering, 64*(4-B), 1899.
9. *Ford, J. D., Steinberg, K. L., Hawke, J., Levine, J., & Zhang, W. (2012). Randomized trial comparison of emotion regulation and relational psychotherapies for PTSD with girls involved in delinquency. *Journal of Clinical Child and Adolescent Psychology, 41*(1), 27-37. doi:[http://dx.doi.org/10.1080/15374416.2012.632343](https://dx.doi.org/10.1176/appi.ajp.2018.18030321)
10. *Fung, J., Guo, S., Jin, J., Bear, L., & Lau, A. (2016). A pilot randomized trial evaluating a school-based mindfulness intervention for ethnic minority youth. *Mindfulness, 7*(4), 819-828. doi:[http://dx.doi.org/10.1007/s12671-016-0519-7](https://dx.doi.org/10.1080/15374416.2011.546044)
11. *Fung, J., Kim, J. J., Jin, J., Chen, G., Bear, L., & Lau, A. S. (2018). A randomized trial evaluating school-based mindfulness intervention for ethnic minority youth: Exploring mediators and moderators of intervention effects. Journal of Abnormal Child Psychology, No Pagination Specified. doi:http://dx.doi.org/10.1007/s10802-018-0425-7
12. Graves, S. L., Herndon-Sobalvarro, A., Nichols, K., Aston, C., Ryan, A., Blefari, A., . . . Prier, D. (2017). Examining the effectiveness of a culturally adapted social-emotional intervention for African American males in an urban setting. School Psychology Quarterly, 32(1), 62-74. doi:https://dx.doi.org/10.1037/spq0000145
13. Goldstein, T. R., Fersch-Podrat, R. K., Rivera, M., Axelson, D. A., Merranko, J., Yu, H., . . . Birmaher, B. (2015). Dialectical behavior therapy for adolescents with bipolar disorder: Results from a pilot randomized trial. Journal of Child and Adolescent Psychopharmacology, 25(2), 140-149. doi:http://dx.doi.org/10.1089/cap.2013.0145
14. *Griffiths, H., Duffy, F., Duffy, L., Brown, S., Hockaday, H., Eliasson, E., . . . Schwannauer, M. (2019). Efficacy of Mentalization-based group therapy for adolescents: the results of a pilot randomised controlled trial. *BMC Psychiatry, 19*(1), 167. doi:[https://dx.doi.org/10.1186/s12888-019-2158-8](http://dx.doi.org/10.1007/s10802-015-9975-0)
15. *Hancock, K. M., Swain, J., Hainsworth, C. J., Dixon, A. L., Koo, S., & Munro, K. (2018). Acceptance and Commitment Therapy versus Cognitive Behavior Therapy for Children With Anxiety: Outcomes of a Randomized Controlled Trial. *Journal of Clinical Child & Adolescent Psychology, 47*(2), 296-311. doi:10.1080/15374416.2015.1110822
16. *Hoorelbeke, K., Koster, E. H. W., Vanderhasselt, M.-A., Callewaert, S., & Demeyer, I. (2015). The influence of cognitive control training on stress reactivity and rumination in response to a lab stressor and naturalistic stress. *Behaviour research and therapy, 69*, 1-10. doi:[https://doi.org/10.1016/j.brat.2015.03.010](https://dx.doi.org/10.2196/11349)
17. *Idsoe, T., Keles, S., Olseth, A. R., & Ogden, T. (2019). Cognitive behavioral treatment for depressed adolescents: results from a cluster randomized controlled trial of a group course. *BMC Psychiatry, 19*(1), 155. doi:[https://dx.doi.org/10.1186/s12888-019-2134-3](https://dx.doi.org/10.1111/eip.12848)
18. *Jacobs, R., Watkins, E., Peters, A., Feldhaus, C., Barba, A., Carbray, J., & Langenecker, S. (2016). Targeting Ruminative Thinking in Adolescents at Risk for Depressive Relapse: rumination-Focused Cognitive Behavior Therapy in a Pilot Randomized Controlled Trial with Resting State fMRI*11*(11), e0163952. Retrieved from [http://cochranelibrary-wiley.com/o/cochrane/clcentral/articles/098/CN-01382098/frame.html](http://dx.doi.org/10.1080/15374416.2012.632343) doi:10.1371/journal.pone.0163952
19. *Kaczkurkin, A. N., Asnaani, A., Zhong, J., & Foa, E. B. (2016). The moderating effect of state anger on treatment outcome in female adolescents with PTSD*. *Journal of Traumatic Stress, 29*(4), 325-331. doi:[http://dx.doi.org/10.1002/jts.22116](https://dx.doi.org/10.1177/0145445517734354)
20. *Kennedy, S. M., Bilek, E. L., & Ehrenreich-May, J. (2018). A Randomized Controlled Pilot Trial of the Unified Protocol for Transdiagnostic Treatment of Emotional Disorders in Children. *Behavior Modification*, 145445517753940. doi:[https://dx.doi.org/10.1177/0145445517753940](http://dx.doi.org/10.1016/j.brat.2015.07.009)
21. *Lackner, N., Unterrainer, H. F., Skliris, D., Shaheen, S., Dunitz-Scheer, M., Wood, G., . . . Neuper, C. (2016). EEG neurofeedback effects in the treatment of adolescent anorexia nervosa. *Brunner-Mazel Eating Disorders Monograph Series, 24*(4), 354-374. doi:[https://dx.doi.org/10.1080/10640266.2016.1160705](https://doi.org/10.1016/j.brat.2015.03.010)
22. *Lee, E. B., Homan, K. J., Morrison, K. L., Ong, C. W., Levin, M. E., & Twohig, M. P. (2020). Acceptance and Commitment Therapy for Trichotillomania: A Randomized Controlled Trial of Adults and Adolescents. Behavior Modification, 44(1), 70-91. doi:https://dx.doi.org/10.1177/0145445518794366
23. *Lindqvist, K., Mechler, J., Carlbring, P., Lilliengren, P., Falkenstrom, F., Andersson, G., . . . Philips, B. (2020). Affect-Focused Psychodynamic Internet-Based Therapy for Adolescent Depression: Randomized Controlled Trial. *Journal of Medical Internet Research, 22*(3), e18047. doi:[https://dx.doi.org/10.2196/18047](https://dx.doi.org/10.1186/s12888-019-2134-3)
24. *Luby, J. L., Barch, D. M., Whalen, D., Tillman, R., & Freedland, K. E. (2018). A Randomized Controlled Trial of Parent-Child Psychotherapy Targeting Emotion Development for Early Childhood Depression. *American Journal of Psychiatry*, appiajp201818030321. doi:[https://dx.doi.org/10.1176/appi.ajp.2018.18030321](http://dx.doi.org/10.1007/s12671-016-0519-7)
25. *McIndoo, C. C., File, A. A., Preddy, T., Clark, C. G., & Hopko, D. R. (2016). Mindfulness-based therapy and behavioral activation: A randomized controlled trial with depressed college students. *Behaviour research and therapy, 77*, 118-128. doi:[https://doi.org/10.1016/j.brat.2015.12.012](https://dx.doi.org/10.1177/0145445517753940)
26. *Mogoaşe, C., Brăilean, A., & David, D. (2013). Can Concreteness Training Alone Reduce Depressive Symptoms? A Randomized Pilot Study Using an Internet-Delivered Protocol. *Cognitive therapy and research, 37*(4), 704-712. doi:10.1007/s10608-012-9514-z
27. *Olson, R. L. (2018). Exercise as a neurobehavioral therapy for cognitive control deficits in major depressive disorder. *Dissertation Abstracts International: Section B: The Sciences and Engineering, 78*(7-B(E)), No Pagination Specified.
28. *Payne, L. (2019). The role of family intervention in improving individual and family functioning in DBT for adolescents. *Dissertation Abstracts International: Section B: The Sciences and Engineering, 79*(7-B(E)), No Pagination Specified.
29. *Schuppert, H., Giesen-Bloo, J., van Gemert, T. G., Wiersema, H. M., Minderaa, R. B., Emmelkamp, P. M., & Nauta, M. H. (2009). Effectiveness of an emotion regulation group training for adolescents-A randomized controlled pilot study. *Clinical Psychology & Psychotherapy, 16*(6), 467-478. doi:[http://dx.doi.org/10.1002/cpp.637](https://dx.doi.org/10.1007/s10802-017-0319-0)
30. Schuppert, H., Timmerman, M. E., Bloo, J., van Gemert, T. G., Wiersema, H. M., Minderaa, R. B., . . . Nauta, M. H. (2012). Emotion regulation training for adolescents with borderline personality disorder traits: A randomized controlled trial. *Journal of the American Academy of Child & Adolescent Psychiatry, 51*(12), 1314-1323. doi:[http://dx.doi.org/10.1016/j.jaac.2012.09.002](http://cochranelibrary-wiley.com/o/cochrane/clcentral/articles/098/CN-01382098/frame.html)
31. *Schweizer, S., Samimi, Z., Hasani, J., Moradi, A., Mirdoraghi, F., & Khaleghi, M. (2017). Improving cognitive control in adolescents with post-traumatic stress disorder (PTSD). *Behaviour research and therapy, 93*, 88-94. doi:[http://dx.doi.org/10.1016/j.brat.2017.03.017](http://dx.doi.org/10.1002/jts.22116)
32. *Shabani, M. J., Mohsenabadi, H., Omidi, A., Lee, E. B., Twohig, M. P., Ahmadvand, A., & Zanjani, Z. (2019). An Iranian study of group acceptance and commitment therapy versus group cognitive behavioral therapy for adolescents with obsessive-compulsive disorder on an optimal dose of selective serotonin reuptake inhibitors. *Journal of Obsessive-Compulsive and Related Disorders, 22*, 100440. doi:[https://doi.org/10.1016/j.jocrd.2019.04.003](https://dx.doi.org/10.1186/s12888-019-2158-8)
33. *Smith, P., Scott, R., Eshkevari, E., Jatta, F., Leigh, E., Harris, V., . . . Yule, W. (2015). Computerised CBT for depressed adolescents: Randomised controlled trial. *Behaviour research and therapy, 73*, 104-110. doi:[http://dx.doi.org/10.1016/j.brat.2015.07.009](https://dx.doi.org/10.1080/10640266.2016.1160705)
34. Suveg, C., Jones, A., Davis, M., Jacob, M. L., Morelen, D., Thomassin, K., & Whitehead, M. (2018). Emotion-Focused Cognitive-Behavioral Therapy for Youth with Anxiety Disorders: A Randomized Trial. *Journal of Abnormal Child Psychology, 46*(3), 569-580. doi:[https://dx.doi.org/10.1007/s10802-017-0319-0](https://dx.doi.org/10.2196/18047)
35. Talley, D. J. (2013). A dismantling study of dialectical behavior therapy for mindfulness with emotionally disturbed adolescents in a residential treatment facility. *Dissertation Abstracts International: Section B: The Sciences and Engineering, 74*(4-B(E)), No Pagination Specified.
36. *Topper, M., Emmelkamp, P., Watkins, E., & Ehring, T. (2017). Prevention of anxiety disorders and depression by targeting excessive worry and rumination in adolescents and young adults: a randomized controlled trial*90*, 123-136. Retrieved from [http://cochranelibrary-wiley.com/o/cochrane/clcentral/articles/675/CN-01297675/frame.html](http://dx.doi.org/10.1016/j.jaac.2012.09.002) doi:10.1016/j.brat.2016.12.015
37. *Webster-Stratton, C. H., Reid, M. J., & Beauchaine, T. (2011). Combining parent and child training for young children with ADHD. *Journal of Clinical Child & Adolescent Psychology, 40*(2), 191-203. doi:[https://dx.doi.org/10.1080/15374416.2011.546044](http://dx.doi.org/10.1016/j.brat.2017.03.017)
38. *Whiteside, U. (2011). A brief personalized feedback intervention integrating a motivational interviewing therapeutic style and dialectical behavioral therapy skills for depressed or anxious heavy drinking young adults. *Dissertation Abstracts International: Section B: The Sciences and Engineering, 71*(12-B), 7745.
39. *Wilkinson, P. O., & Goodyer, I. M. (2008). The effects of cognitive-behavioural therapy on mood-related ruminative response style in depressed adolescents. *Child and Adolescent Psychiatry and Mental Health, 2*(1), 3. doi:10.1186/1753-2000-2-3
40. *Wineman, P. A. (2009). The efficacy of a dialectical behavior therapy-based journal-writing group with inpatient adolescent females: Improving emotion regulation, depressive symptoms and suicidal ideation. *Dissertation Abstracts International: Section B: The Sciences and Engineering, 70*(6-B), 3817.
41. *Yang, W., Zhang, J. X., Ding, Z., & Xiao, L. (2016). Attention Bias Modification Treatment for Adolescents With Major Depression: A Randomized Controlled Trial. *Journal of the American Academy of Child & Adolescent Psychiatry, 55*(3), 208-218.e202. doi:10.1016/j.jaac.2015.12.005
